# Formaldehyde Exposure and Acute Myeloid Leukemia: A Review of the Literature

**DOI:** 10.3390/medicina55100638

**Published:** 2019-09-25

**Authors:** Alessandro Allegra, Giovanna Spatari, Stefano Mattioli, Stefania Curti, Vanessa Innao, Roberta Ettari, Andrea Gaetano Allegra, Concetto Giorgianni, Sebastiano Gangemi, Caterina Musolino

**Affiliations:** 1 Division of Hematology, Department of Human Pathology in Adulthood and Childhood, University of Messina, 98122 Messina, Italy; vinnao@unime.it (V.I.); andrea.allegra@hotmail.it (A.G.A.); cmusolino@unime.it (C.M.); 2 Department of Environmental Science, Safety, Territory, Food and Health, University of Messina, 98122 Messina, Italy; gspatari@unime.it (G.S.); mariogiorgianni@virgilio.it (C.G.); 3 Department of Medical and Surgical Sciences (DIMEC), University of Bologna, 40126 Bologna, Italy; smattioli@unibo.it (S.M.); stefania.curti@unibo.it (S.C.); 4 Department of Chemical, Biological, Pharmaceutical and Environmental Sciences, University of Messina, 98122 Messina, Italy; rettari@unime.it; 5 School and Division of Allergy and Clinical Immunology, Department of Clinical and Experimental Medicine, University Hospital “G. Martino”, Via Consolare Valeria SNC, 98125 Messina, Italy; sgangemi@unime.it

**Keywords:** formaldehyde, acute myeloid leukemia, occupational exposure, cancer, carcinogenesis

## Abstract

*Background and objective:* The aim of the present study was to evaluate associations between cumulative and peak formaldehyde exposure and occurrence of acute myeloid leukemia. *Material and Methods:* A comprehensive search was performed using the PubMed and Embase databases. We included studies presenting information about the role of formaldehyde in leukemic occurrence and mortality risk. Then, full texts of the selected references were assessed, and references of included studies were checked to identify additional articles. *Result:* The information was then summarized and organized in the present review. A total of 81 articles were obtained from the search. *Conclusion:* Findings from the review of the literature do not support the hypothesis that formaldehyde is a cause of acute myeloid leukemia.

## 1. Introduction

Formaldehyde (FA) is an organic compound with the formula CH_2_O. It is the simplest of the aldehydes. The safety of formaldehyde is very problematic. It is not acutely toxic, as ingestion of many milliliters is tolerated. However, FA could have wide ranging effects in disease states, particularly cancer progression [1]. FA has displayed mutagenic and genotoxic actions in several experimental models in vivo and in vitro [2].

Work-related contact with FA happens in a multiplicity of diverse areas, although employees at greater risk are those engaged in the healthcare area and in wood treating manufacturing [3]. Consumer products comprising personal care goods, cigarette smoking, some medicines, and environmental pollution are common nonoccupational sources [4]. Finally, FA exposure also has endogenous fonts. In fact, it is generated intracellularly as a constituent of the one carbon group intermediate metabolism pathway.

FA is classified as a human carcinogen, and its connection to nasopharyngeal cancer has been demonstrated in several epidemiological reports [5]. Moreover, in 2009, the International Agency for Research on Cancer Working Group concluded that there is suggestion for the carcinogenicity of FA in humans [6].

In 2010, the US Environmental Protection Agency (EPA) also concluded an evaluation of the existing proof for the carcinogenicity of FA and established that epidemiological support is adequate to affirm a causative correlation between FA exposure and nasopharyngeal cancer, all leukemias, and lympho-hematopoietic diseases. Nevertheless, a commission organized by the National Research Council established that the EPA 2010 valuation did not offer a sure basis for causal association [7]. Moreover, the Nuclear Regulatory Commission (NRC) settled that the research of a causative basis for these diseases is difficult, considering the contradictions in the epidemiologic results and faint animal data [8].

Furthermore, evaluations of updated mortality for the National Cancer Institute cohort stated connections of “peak” exposures with acute myeloid leukemia (AML), but not with cumulative, median, or rate of “peak” exposures [9].

Finally, AML and chronic myeloid leukemia (CML) were described together in the NCI reports, and were mingled as Myeloid Leukemias (ML). However, though both CML and AML result from myeloid stem cells, the risk elements connected with AML and CML vary, and these diversities cause the question of whether the connection between FA exposure and merged MLs displays a real association between FA exposure and AML.

## 2. Materials and Methods

A comprehensive search was accomplished employing PubMed and the following query (date of last search, 28 May, 2018): (#1 Leukemia, Myeloid, Acute [MH] OR Precursor Cell Lymphoblastic Leukemia-Lymphoma [MH] #2 formaldehyde OR “Methyl aldehyde” OR “formic aldehyde” OR formalin OR formol OR methanol #3, #1 AND #2). We found 72 papers. Moreover, we made a research using Embase and the following query (date of last search, 22 May, 2018): (#1 Leukemia, Myeloid, Acute [MH] OR Precursor Cell Lymphoblastic Leukemia Lymphoma [MH] #2 formaldehyde OR “Methyl aldehyde” OR “formic aldehyde” OR formalin OR formol OR methanol #3, #1 AND #2). We found 77 papers. A total of 81 articles were obtained from the searches. In the first phase, titles and abstracts were examined by two researchers, according to precise criteria stated, in order to regulate this systematic review. Any divergence was decided by a third, independent researcher. We included papers presenting data about the role of FA in acute leukemic pathogenesis, occurrence, and mortality risk. In the next phase, full texts of the selected articles were evaluated, and references of included papers were examined to find further works.

The information was then summarized and organized in the present review.

## 3. Results

Checkoway et al. valued the results contained in the update of the NCI cohort [10]. They replicated the data stated by Beane Freeman et al. [9] and performed supplementary investigations about the possible relations of AML with peak exposure, using a different, more traditional definition of peak (Table 1).

A total of 25,619 laborers were monitored from the year of first engagement at the facility (1930 to 1966) or year of cohort identification (1934 to 1958), through death, loss-to-follow-up, or 31 December 2004. They evaluated 997,514 person–years. Of the 25,619 employers, 3478 (13.6%) operated in employment with peaks of 2 ppm or more, to less than 4 ppm, and 2907 (11.3%) had works with peaks of 4 ppm or more.

No correlation between cumulative FA exposure and death from all types of leukemia was detected for the total cohort. Nevertheless, risks were increased among those working for one year or more, unrelatedly to total the exposure group, due to the great loss of leukemia cases in the referent class (27 subjects labored less than one year)–hazard ratio (HR) = 2.44; 95% CI, 1.08 to 5.51 for subjects with total exposures of 0.5 to less than 2.5 ppm–years and HR = 2.49; 95% CI, 1.13 to 5.49 for subjects with 2.5 ppm–years or more (P_trends_ = 0.04). Peak exposure 2.0 ppm or more, to less than 4 ppm (HR = 2.46; 95% CI, 1.29 to 4.67 and HR = 2.45; 95% CI, 1.32 to 4.52, respectively).

ML (all forms) was not correlated with total FA exposure in the complete cohort. There was, nevertheless, a not statistically significant connection of total exposure and AML among labors working one year or more. Peak exposure of 2.0 ppm or more, to less than 4 ppm was correlated with AML in the total cohort (HR = 2.09; 95% CI, 1.03 to 4.26) and analogously among subjects working one year or more (HR = 2.49, 95% CI, 1.01 to 6.15). HR for peaks of 4.0 ppm or more were weaker, but still increased, and trends were not statistically significant (P_trend_ = 0.06 and 0.08 respectively).

Then, agreeing with their data, AML was unconnected to total exposure.

Nevertheless, the study has numerous limits. The first is that work positions were not verified beyond the first study end date; therefore, exposures could not be calculated for years worked after 1980. Furthermore, notwithstanding almost one million person–years of follow up, there were a slight number of AML deaths detected among subjects who were engaged for more than one year and most greatly exposed to FA. Moreover, few of the subjects who died of AML had any peak exposures, and almost none had peak exposures within an acceptable time window of latency.

In a diverse report, authors described the data from research planned to verify if FA exposure disturbs hematopoietic action and causes leukemia-related chromosome modifications in Chinese employees [11]. This research involved 94 workers, with 51 health subjects used as controls and 43 exposed to FA in the workstation. Biological samples were taken from the blood and urine of the workers. For the exposed employees, samples were gathered after employees had been examined at least twice for FA air exposure in the workplace. A survey was also dispensed to attain data on medical and occupational history, environmental exposures, recent medicines and alcohol and tobacco consumption. Routine blood and biochemical tests of the biological samples were performed, as well as culturing of peripheral blood cells.

They described statistically significant reduction in red blood cell (RBC), platelet, white blood cell (WBC), and lymphocyte amounts, with statistically significant augments in RBC mean corpuscular volume in FA exposed employees compared to healthy subjects. Moreover, a 20% reduction in mean colony formation occurrence was described for the CFU-GM progenitor cells in exposed employees compared with those in healthy subjects, which Zhang proposed as a probable inhibitory action of FA on myeloid progenitor cells. The FISH investigation of the CFU-GM colonies was restricted to 10 highly exposed workers (median exposure of 2.14 parts per million FA) and was compared to 12 control samples. The researchers described an augmented occurrence of monosomy 7 and trisomy 8 in metaphase spreads obtained from cultures of CFU-GM colony cells [11]. According to their data, Zhang et al. settled that FA exposure can cause unfavorable action on the hematopoietic system, and that leukemia initiation by FA is biologically possible, although authors discussed the limitations of their research and the need for replication and extension of the study [12].

Actually, a serious review of the accessible data from this work has recognized several methodological inadequacies [13]. Precisely, the statement that aneuploidy of some chromosomes occurred in vivo in hematopoietic stem cells in human subjects exposed to FA has not been proved. If we consider the kinetics of CFU-GM colony formation, the aneuploidy assessed could not have risen in vivo, but had to have happened throughout cell culture in vitro. Reanalysis of the results does not sustain a causal connection between FA and AML. Moreover, in a later study, Mundt et al. performed a linear regression analysis on Zhang’s study. Results showed that differences in white blood cell, granulocyte, platelet, and red blood cell counts were not exposure dependent. Among formaldehyde-exposed workers, no association was observed between individual average formaldehyde exposure estimates and frequency of aneuploidy [14].

Then, Gentry et al. found no constant statistically significant association between FA exposure and chromosome aberrations [13]. Sister chromatid exchanges (SCEs) or micronucleated cells in hematopoietic stem cells have not been observed in in vivo tests or in animal models.

However, in a study, the frequencies of micronuclei in nasal mucosa cells and SCEs of peripheral lymphocytes were assessed in 18 non-smoking workers (mean exposure duration 8.6 years) in an FA factory and 16 non-smoking waiters exposed to FA for 12 weeks in a ballroom. A non-smoking group without occupational contact (n = 23) to FA was employed as a control. The 8 h time-weighted average (TWA) concentrations of formaldehyde were 0.985+/−0.286 mg/m^3^ with a ceiling exposure concentration of 1.694 mg/m^3^ in the workshop, and 0.107+/−0.067 mg/m^3^ in the ballroom (5 h TWA). Higher frequencies of micronuclei per thousand cells in nasal mucosa cells of workers versus the control (2.70+/−1.50 versus 1.25+/−0.65, *p* < 0.05) and higher frequency of SCEs in peripheral lymphocytes of workers group (8.24+/−0.89 versus 6.38+/−0.41, *p* < 0.05) were observed. Nevertheless, these cells were neither hematopoietic stem cells nor physiological substitutes for hematopoietic stem cells [15].

In a diverse work, Jones et al. created a Job Exposure Matrix (JEM) and recreated the possible exposures to several substances for six employees’ diagnosed tumors (three AML, two Acute Lymphoblastic Leukemia, one Non-Hodgkin Lymphoma) [16]. With this JEM, the amount of the potential cumulative contact was recognized. Two diverse approaches were employed to assess if each worker’s tumor was related to occupational exposure. The first method was to use toxicity values to evaluate the excess lifetime tumor risk related to potential exposure to specific substances. The toxicity employed comprised the inhalation unit risk value derived from the US EPA Integrated Risk Information System and the lifetime attributable cancer risk associated with ionizing radiation from the Biological Effects of Ionizing Radiation model. The second method was to analyze existing epidemiological studies with respect to the risk of analogous tumors described in the semiconductor industry, and the greatness of the workers’ potential exposure relative to exposure concentrations for which excess cancer incidence was reported in epidemiological studies cited by consensus panels or government agencies.

They established that two workers were possibly exposed to factors that have been categorized as known, probable, or possible human carcinogens and related to their specific tumors. However, these employees’ potential exposures to these factors are analogous to outdoor environmental concentrations. In this study, no relationship between workplace exposures to the accepted hematopoietic carcinogens and the occurrence of the workers’ tumors can be confirmed.

Moreover, FA had not been utilized as a process chemical and was not an expected process by-product in these lines. Finally, in this research, hematopoietic diseases were related to peak exposure, but this exposure amount was extremely undefined because of the lack of actual monitoring data of short-term peak exposures.

Lastly, in a critical review of the literature by Charbotel et al., it was settled that indication from meta-analyses sustain the connections of AML with FA [17], although the only data supported is that of Zhang’s work [11].

## 4. Discussion

Lymphomas and leukemias are one of the most frequent forms of tumors caused by a wide multiplicity of tumor-inducing agents. Of the 66 Group 2A factors, there is support, founded either on human investigations or animal models, that at least 10 of these agents are apt to provoke lymphoma or leukemia in human subjects [18].

**Figure 1:**
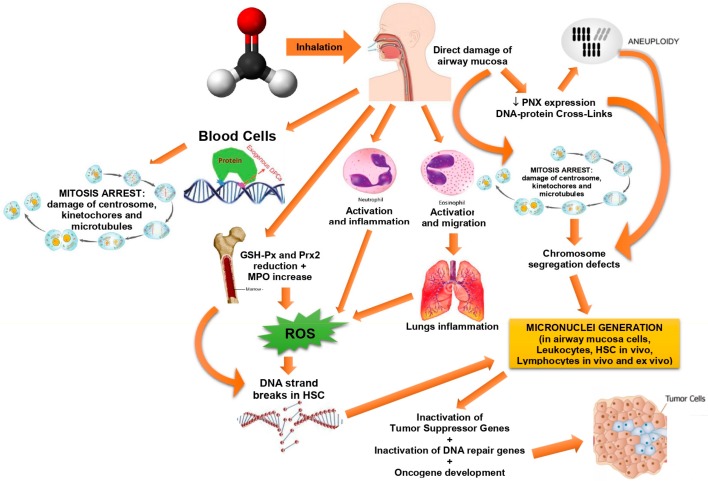
Possible formaldehyde (FA) genotoxic and cytotoxic actions.

FA is a chemical agent that principally binds to sulfhydryl and primary N-terminal amine groups in polypeptides and proteins. FA can bind to DNA, making monoadducts and DNA–DNA crosslinks [19], as well as DNA-protein crosslinks, which have been demonstrated to be related with their genotoxic and cytotoxic actions [20] (Figure 1). FA binds promptly to reduced glutathione (GSH) nonenzymatically, producing an S-hydroxylmethylglutathione, hemi-thioacetal adduct that can be transformed to format in a multi-step reaction by FA dehydrogenase and S-formylglutathione hydrolase, liberating free GSH.

FA has displayed mutagenic and genotoxic actions in several experimental models in vivo and in vitro [2]. In vitro, it has been exhibited to cause mutations in mammalian cells and bacteria, and chromosomal aberrations. In mice models, DNA adducts, micronuclei, and DNA-protein crosslinks have been detected at the site of exposure, but FA-derived DNA adducts have not been seen in the bone marrow (BM) [21,22].

The absence of FA-derived DNA adducts in the BM is probably a sign that a generation of leukemia by FA is not biologically probable [23,24]. Nevertheless, recently, there have been several researches that have registered significant augments in toxicity, oxidative damage, and DNA damage in the bone marrow of mice exposed to FA [25,26] (Table 2). In human subjects, FA-exposed employees have been described to present augmented occurrences of DNA-protein crosslinks and structural chromosome mutations in lymphocyte [27].

According to the data presented in a recent work, it was suggested that leukemia ensuing from exposure to FA could happen by one of the following processes: Direct injury to stem cells in the bone marrow; injury to circulating blood stem cells; or injury to pluripotent stem cells [28]. On the other hand, hematopoietic diseases are commonly believed to have a multifactorial etiology, implicating genetics and environmental factors, and thus FA exposure alone is probably incapable of provoking leukemia.

## 5. Conclusions

Numerous previous analyses producing variable findings have been presented, which may have resulted from diverse approaches, showing the heterogeneity of contact and validity among published studies. In our review, we presented both epidemiological studies and toxicological and mechanistic evidences. Our analysis of the data from the literature provides no support for the hypothesis that formaldehyde causes acute leukemia. In fact, with the exception of Zhang’s work [11], the studies we have examined and placed in our review do not appear to reveal a causative correlation between exposure to FA and acute leukemia. A recent review of the epidemiological evidence regarding the relationships between formaldehyde exposure and cancer identified causal relationships and significant dose–response relationships between FA contact and nasopharyngeal cancer and lymphoma, but, although lymphohematopoietic malignancies, including leukemia, should be considered in cases with occupational formaldehyde exposure, additional evidence is needed to confirm the relationship between formaldehyde exposure and cancer [29].

However, our review has some limitations. There are limited papers about the action of FA in human subjects, and some contain a restricted number of patients and follow a cross-sectional design. Moreover, most researches included present methodologic bias.

Alternative explanations for associations seen between the combined groups of exposed and unexposed workers will have to be explored, while the questionable inferential value of the monosomy 7 and trisomy 8 prevalence findings, based on methods deviating from protocol, will also need to be addressed [30]. Future studies on the carcinogenic action of FA will probably make use of novel sensing systems that could be suitable to real-time environmental and workplace monitoring [31]. These new methods may also make the analysis of the existence of a dose–response correlation more certain. Finally, more studies in large prospective cohorts are needed to confirm if FA exposure may affect the development of acute leukemia.

## Figures and Tables

**Table 1:** Main characteristics of studies included in the present review.

Authors	Years	Type of Study	Limitations	Conclusions
Checkoway et al.	2015	Cohort study	Job assignments were not documented beyond the initial study end date. There is a relatively small number of AML deaths observed among individuals employed for more than 1 year and most highly exposed to formaldehyde. Few of the employees who died of AML had any peak exposures, and nearly none had peak exposures within a reasonable time window of latency	No association
Zhang et al.	2010	Molecular epidemiology study	Methodological limitations	Leukemia induction by formaldehyde is biologically plausible
Gentry et al.	2013	Reanalysis of the data		No association
Jones et al.	2015	Quantitative risk assessment	Formaldehyde had not been used as a process chemical and was not an expected process by-product in these lines. Exposure measure was highly uncertain because of the absence of actual monitoring data of short-term peak exposures	No association
Charbotel et al.	2014	Review of the literature	Only data supported is those of Zhang’s work	Association

**Table 2:** Main characteristics of studies on animal models.

Authors	Year	Animal Model	Conclusions
Moeller et al.	2011	Cynomolgus macaques	Endogenous N(2)-hydroxymethyl-dG adducts in DNA were found in the bone marrow of animals exposed to 1.9 and 6.1 ppm of [(13)CD(2)]- formaldehyde for 6 h a day for 2 consecutive days
Lu et al.	2011	Rats	Exogenous adducts were not detectable in the bone marrow of rats exposed to 15.2 ppm [(13)CD(2)] of formaldehyde.
Katnelson et al.	2013	Rats	Inhalation exposure to formaldehyde vapors (12.8 ± 0.69 mg/m^3^) 4 h per day, 5 days per week during 10 weeks, induced changes in differential WBC count and bone marrow micronuclei count.
Ji et al.	2014	Cultured mouse hematopoietic stem/progenitor cells	FA significantly induced micronuclei in mice in erythroid progenitor cells
